# Diabetes mellitus and the risk of gastric cancer: a meta-analysis of cohort studies

**DOI:** 10.18632/oncotarget.16487

**Published:** 2017-03-22

**Authors:** Zhi-Feng Miao, Hao Xu, Ying-Ying Xu, Zhen-Ning Wang, Ting-Ting Zhao, Yong-Xi Song, Hui-Mian Xu

**Affiliations:** ^1^ Department of Surgical Oncology, First Hospital of China Medical University, Shenyang, Liaoning Province, China; ^2^ Department of Breast Surgery, First Hospital of China Medical University, Shenyang, Liaoning Province, China

**Keywords:** gastric cancer, diabetes mellitus, meta-analysis, gender, prognosis

## Abstract

Studies examining the relationship between diabetes mellitus (DM) and the risk of gastric cancer incidence or gastric cancer mortality have produced inconsistent results. The purpose of this study was to evaluate the evidence regarding the relationship between DM and subsequent gastric cancer incidence or gastric cancer mortality risk on the basis of cohort studies. A systematic search of articles in PubMed, EmBase, the Cochrane Library, and reference lists was conducted to identify relevant literature. Twenty-two cohort studies reporting data on 8,559,861 participants were included in the study. Overall, participants with DM had little or no change in the risk of gastric cancer, or gastric cancer mortality. There was no evidence of difference in the RR for gastric cancer between men and women. Participants with DM had a non-significant trend towards an increased risk of gastric cancer mortality in men. There was no significant difference between men and women for this relationship. Finally, although subgroup analysis suggested DM was associated with a significant impact on gastric cancer incidence and gastric cancer mortality risk in several specific populations, a significance based on gender difference was not observed. In conclusion, DM might increase the risk of gastric cancer in men when the study used standard incidence/mortality ratio as effect estimate. Further, DM were associated with higher risk of gastric cancer mortality in men if the mean age at baseline less than 55.0 years, used RR or HR as effect estimate, the study adjusted smoking or not, and the study not adjusted alcohol drinking.

## INTRODUCTION

Diabetes mellitus (DM) is a growing global pandemic afflicting approximately three to four percent of adults worldwide. An estimated 366 million people worldwide will development in DM by 2030 [1–4]. DM may predispose patients to premature illness and death due to the relevant risk of cardiovascular diseases [5–6]. In addition, the relationship between DM and cancer risk has been examined in numerous meta-analyses [7–14]. Epidemiologic studies examining the association between DM and gastric cancer risk have largely been inconclusive and provide conflicting results [15–21], including two meta-analyses of the relationship between DM and the risk of gastric cancer [22–23]. Furthermore, whether these relationships differ according to gender in specific subpopulations remains controversial.

In 2012 alone, there were approximately 952,000 gastric cancer cases and 723,000 deaths from gastric cancer worldwide, accounting for 6.8% of the total cancer cases and 8.8% of all cancer deaths [24]. Several meta-analyses have indicated that numerous lifestyle factors might play beneficial or harmful impacts on the risk of gastric cancer [25–27]. Yang et al suggested that being overweight or obese associates with an increased risk of gastric cancer and the strength of this relationship increases with increasing body mass index (BMI) [28]. It is also worth noting that increased BMI is associated with an increased risk of DM [29]. Clarifying the potential role that DM plays on the risk of gastric cancer is particularly important in the DM populations, as it has not been definitively determined. Hence, the role of DM on the risk of gastric cancer incidence or mortality still needs further evaluation and discussion. Here we attempted a large-scale examination of the available cohort studies to determine the association between DM and the incidence of gastric cancer or gastric cancer mortality. Furthermore, we also evaluated gender differences in this relationship in patients with different baseline characteristics.

## RESULTS

### Literature search

**Figure 1:**
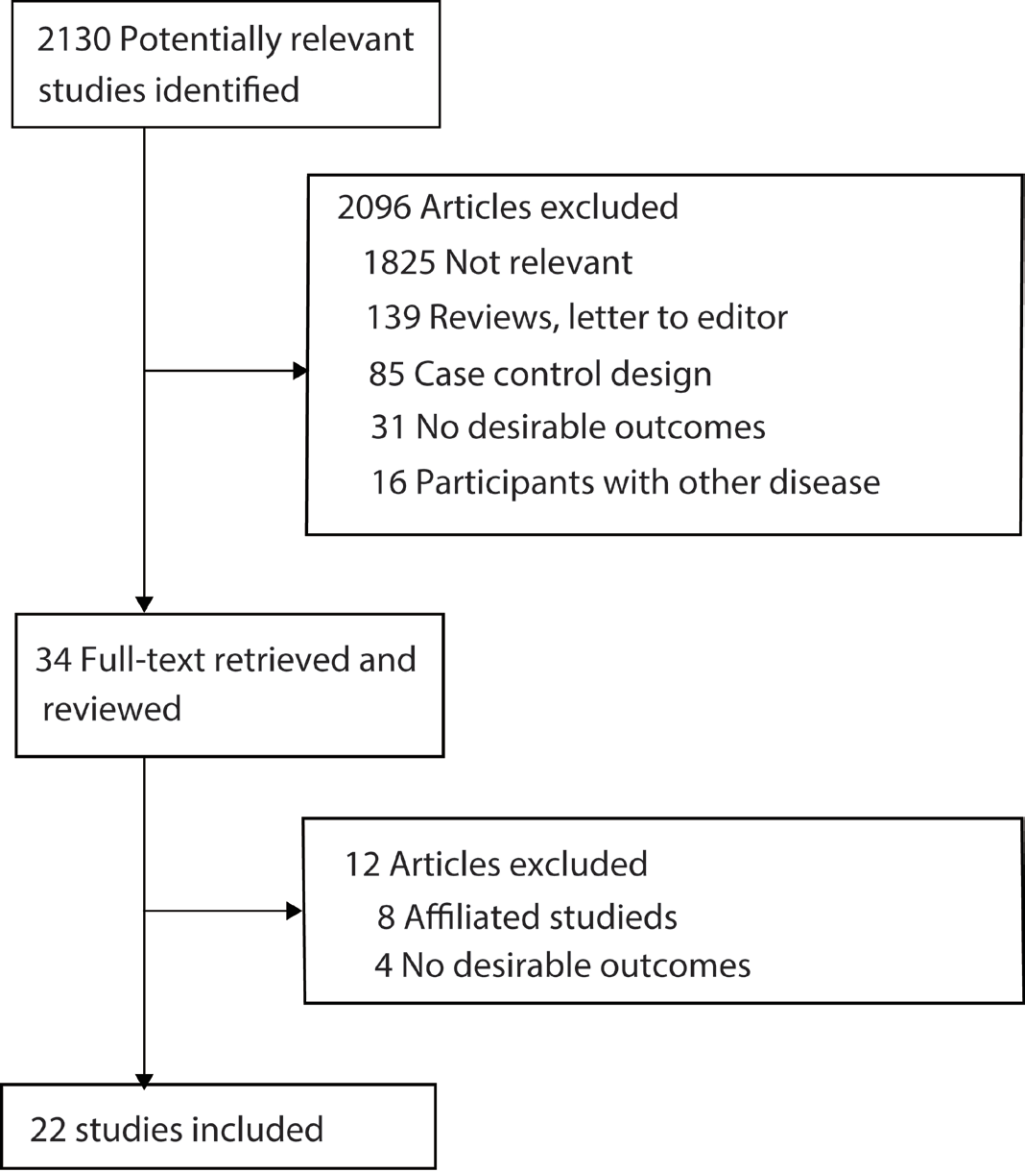
Flow diagram of the literature search and trials selection process

The process of study selection is presented as a flow chart in Figure 1. A total of 2,130 articles from the initial search were identified and screened, of which 2096 were excluded due to being irrelevant, reviews, letters to the editor, having a case control design, producing no desirable outcomes, or including participants with other diseases. A total of 34 studies were reviewed in detail, and 4 without gastric cancer incidence or mortality outcomes were ruled out and another 8 studies were excluded as being different publications of the same sample of participants, thereby including main results that had already been reviewed [30–37]. Ultimately, 22 studies were eligible for the final pooled analysis [15–21, 49–63]. A manual search of the reference lists of these studies did not yield any new eligible studies. The general characteristics of the included studies are presented in Table 1.

**Table 1:** Baseline characteristics of studies included in the systematic review and meta-analysis

Study	Place	Assessment of exposure	Sample size	Age at baseline	Gender (M/F)	Percentage of overweight (%)	Gastric cancer cases	Death due to gastric cancer cases	Effect estimate	Follow-up (year)	Adjusted factors	NOS score
Wideroff L 1997 [15]	Denmark	Danish Cancer Registry	109581	64.0 for men and 69.0 for women	54571/55010	NA	319	NA	SIR	17.0	Age, sex, calendar year	6
CPS II 2004 [49]	USA	Self administered questionnaire	1056243	57.0	467922/588321	48.9	NA	109	RR	12.5	Age, sex, race, education, family history, BMI, PA, smoking, alcohol, diet	8
NHIC 2005 [16]	Korea	Self-report and blood glucose levels	1298385	47.0	829770/468615	NA	1120	511	HR	10.0	Age, smoking, alcohol	9
JPHC 2006 [50]	Japan	Self-report	97771	51.4 for men and 51.8 for women	46548/51223	27.3	1339	NA	HR	14.0	Age, study area, VD, smoking, alcohol, BMI, PA, vegetable and coffee intake	8
MHS 2010 [51]	Israel	Self-report or blood glucose level	100595	61.6	52913/47682	79.4	307	NA	HR	8.0	Age, region, SES level, use of healthcare services, BMI, and history of VD	7
Hemminki K 2010 [17]	Sweden	Medical records	125126	>39.0	NA	NA	469	NA	SIR	15.0	NA	5
NIH-AARP Diet and Health Study 2011 [18]	USA	Self-report	469448	62.0	280883/188565	64.6	631	NA	HR	10.0	Age, sex, calories, alcohol, smoking, fruit consumption, ethnicity, education, and PA	8
Tseng CH 2011 [52]	Taiwan	Self-report	88694	>25.0	40799/47895	NA	NA	1049	SMR	12.0	Age and sex	6
Verona Diabetes Study 2003 [53]	Italy	Medical records	7148	67.0	3366/3782	70.7	NA	48	SMR	10.0	Age, smoking, BMI	7
Swerdlow AJ 2005 [54]	UK	Self-report	5066	30-49	2944/2122	NA	12	9	SIR	18.0	Age, sex, calendar year, residence	6
Kessler II 1970 [55]	USA	Blood glucose test	218313	40-59	96010/122313	NA	NA	98	SMR	10.0	Age, sex	6
HIC 2009 [56]	Scotland	Self-report	28731	62.0	15227/13504	NA	NA	62	RR	3.9	Deprivation decile	7
U.S. Veterans 2010 [57]	USA	Discharge diagnosis	4501578	59.1	4501578/0	5.7	7515	NA	RR	10.5	Age, time, latency, race, number of visits,alcohol, obesity and COPD	8
Zendehdel K 2003 [19]	Sweden	Medical records	29187	38.7	14864/14323	NA	10	NA	SIR	14.4	Excluding the 1 st -year of follow-up	7
Koskinen SV 1998 [58]	Finland	Census records	58000	30-74	24000/34000	NA	NA	73	RR	5.0	Age	6
JACC 2006 [20]	Japan	Self administered questionnaire	56881	40-79	23378/33503	19.8	631	NA	RR	18.0-20.0	Age, BMI, smoking, and drinking	8
Ragozzino M 1982 [59]	USA	Blood glucose levels	1135	61.0	602/533	NA	8	NA	SIR	8.6	Age, sex	7
Adami HO 1991 [21]	Sweden	Medical records	51008	NA	23146/27862	NA	159	NA	RR	5.2	Age, sex	6
Whitehall study 2004 [60]	UK	Oral glucose tolerance test	18006	51.5	18006/0	NA	NA	162	HR	25.0	Age, employment, smoking, SBP, PA, disease history	7
NHIRD 2013 [61]	Taiwan	Medical records	98125	56.0	54675/43450	NA	263	NA	HR	5.5	Age, sex, CGD, pneumoconiosis	8
Strong Heart Study 2015 [62]	USA	Self administered questionnaire	4419	55.1	1794/2625	50.9	NA	19	HR	20.0	Age, sex, center, BMI, education, drinking status and smoking	8
Xu HL 2015 [63]	China	Self administered questionnaire	136421	53.4	61480/74941	NA	755	NA	HR	7.5 for men and 13.2 for women	Age, sex, education, income, BMI, CGD, family history of stomach cancer, PA, EI, smoking, tea, alcohol, vegetable, red meat, and fruit intake	8

Abbreviations: BMI: body mass index; PA: physical activity; VD: vascular disease; EI: energy intake; CGD: chronic gastric disease; NA: not available.

### Study characteristics

The 22 included studies covered a total of 8,559,861 individuals and reported 13,538 new gastric cancer cases and 2,140 deaths due to gastric cancer. The sample size for each individual study was 1,135-4,501,578 participants, while the follow-up period for participants was 3.9-25.0 years. Seven studies were conducted in Asia [16,20, 50–52, 61, 63], 9 in Europe [15, 17, 19, 21, 53, 54, 56, 58, 60], and 6 in the USA [18, 49, 55, 57, 59, 62]. 4 studies used self-administered questionnaires [20, 49, 62, 63], 7 studies used self-reporting [16, 18, 50–52, 54, 56], 7 studies used medical records [15, 17, 19, 21, 53, 58, 61], and the remaining 4 studies used blood glucose tests [55, 57, 59, 60] to assess exposure. Eight studies used SIR/SMR to evaluate the relationship between DM and gastric cancer incidence or mortality [15,1 7, 19, 52–55, 59], and the remaining 14 studies used OR, RR or HR to calculate this association [16, 18, 20, 21, 49-51, 56-58, 60-63]. Fourteen studies evaluated the relationship between DM and the incidence of gastric cancer [15-21, 50, 51, 54, 57, 59, 61, 63], and ten studies evaluated the relationship between DM and the risk of gastric cancer mortality [16, 49, 52-56, 58, 60, 62]. Study quality was evaluated using the NOS score (Table 1) [39]. Overall, 1 study had a score of 9 [16], 8 studies had a score of 8 [18, 20, 49, 50, 57, 61-63], 6 studies had a score of 7 [19, 51, 53, 56, 59, 60], 6 studies had a score of 6 [15, 21, 52, 54, 55, 58], and the remaining 1 study had a score of 5 [17].

### DM and the risk of gastric cancer incidence or mortality

**Figure 2:**
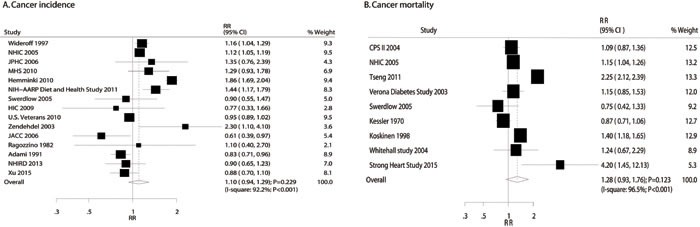
Association of diabetes mellitus with the risk of gastric cancer incidence (**A**) and mortality (**B**).

A total of 15 studies reported an association between DM and the incidence of gastric cancer [15-21, 50, 51, 54, 56, 57, 59, 61, 63]. The summary RR showed that participants with DM were not associated with a change in gastric cancer risk (RR: 1.10; 95%CI: 0.94-1.29; *P* = 0.229; Figure 2A), but substantial heterogeneity was detected (*P* < 0.001). Furthermore, a total of 9 studies reported an association between DM and the risk of gastric cancer mortality [16, 49, 52-55, 58, 60, 62]. There was no significant association between DM patients and participants without DM for gastric cancer mortality across all studies (RR: 1.28; 95%CI: 0.93-1.76; *P* = 0.123; Figure 2B). Substantial heterogeneity was observed in the magnitude of the effect across the studies (*P* < 0.001).

### DM and the risk of gastric cancer incidence in men and women

**Figure 3:**
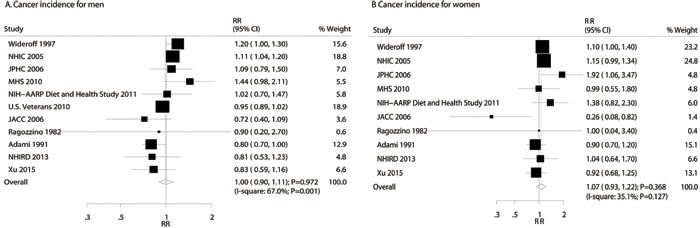
Association of diabetes mellitus with the risk of gastric cancer incidence in men (**A**) and women (**B**).

There were 11 studies with data available for men [15, 16, 18, 20, 21, 50, 51, 57, 59, 61, 63] and 10 studies for women [15, 16, 18, 20, 21, 50, 51, 59, 61, 63]. The summary analysis results for men and women with or without DM showed that DM was not associated with the risk of gastric cancer incidence in men (RR: 1.00; 95%CI: 0.90-1.11; *P* = 0.972; Figure 3A) or in women (RR: 1.07; 95%CI: 0.93-1.22; *P* = 0.368; Figure 3B). Gender difference was not significantly associated with the relationship between DM and gastric cancer incidence (RRR: 0.93; 95%CI: 0.77-1.14; *P* = 0.495). Furthermore, we noted potential evidence of heterogeneity for gastric cancer in men (I-square: 67.0%; *P* = 0.001), and mild heterogeneity for gastric cancer in women (I-square: 35.1%; *P* = 0.127). Once sensitivity analyses were conducted for men and women, we noted that the conclusion was not affected by the systematic exclusion of any one specific study from the pool (Supplemental 2: Tables S1 and S2). However, women were found to have increased risk for gastric cancer when excluding the JACC study [20] (RR: 1.09; 95%CI: 0.99-1.20; P *P* = 0.066; I-square: 0.9%; *P* value for heterogeneity: 0.426), which illustrated the incidence of gastric cancer is gradually decreasing in Japan recently.

### DM and the risk of gastric cancer mortality in men and women

**Figure 4:**
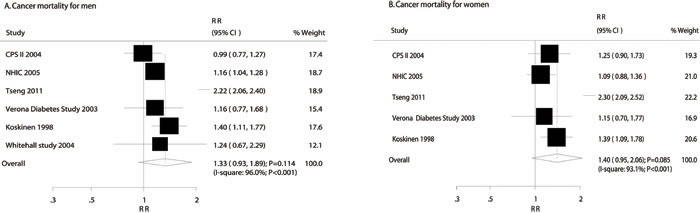
Association of diabetes mellitus with the risk of gastric cancer mortality in men (**A**) and women (**B**).

The breakdown for the number of studies available for mortality associations with men and women was 6 [16, 49, 52, 53, 58, 60] and 5 studies [16, 49, 52, 53, 58], respectively. The summary analysis results for men and women with or without DM indicated that DM was not associated with the risk of gastric cancer mortality in men (RR: 1.33; 95%CI: 0.93-1.89; *P* = 0.114; Figure 4A) or women (RR: 1.40; 95%CI: 0.95-2.06; *P* = 0.085; Figure 4B). The RRR indicated that no gender difference existed for this relationship (RRR: 0.95; 95%CI: 0.56-1.61; *P* = 0.848). Substantial heterogeneity was detected for gastric cancer mortality in men and women (men: I-square: 96.0%, *P* < 0.001; women: 93.1%, *P* < 0.001). According to sensitivity analyses, DM was associated with an increased risk of gastric cancer mortality in men when excluding the Tseng study, the study specific reported SMR at different age stages, and the effect estimate was inconceivable higher in DM patients aged 25-64 years [52] (RR: 1.17; 95%CI: 1.07-1.28; *P* < 0.001; I-square: 1.2%; *P* value for heterogeneity: 0.400; Supplemental 2: Table S3), which specifically included a wide range of participants and had a mean age greater than 25.0 years. Similarly, after excluding the Tseng study [52], we noted participants with DM may have an increased risk of gastric cancer mortality in women (RR: 1.21; 95%CI: 1.06-1.40; *P* = 0.006; I-square: 0.0%; *P* value for heterogeneity: 0.533; Supplemental 2: Table S4).

### Subgroup analysis

Substantial heterogeneity was detected for gastric cancer incidence and mortality in men and women. We therefore performed subgroup analyses to minimize heterogeneity and evaluate the potential role of DM on the progression of gastric cancer among the included studies. First, DM was associated with an increased risk of gastric cancer incidence in men if the study used SIR/SMR as an effect estimate index (Table 2). Second, participants with DM showed an increased risk of gastric cancer mortality if the study was conducted on women in Western countries, the study used OR, RR, or HR as an effect estimate index, and the study was not adjusted for smoking or alcohol consumption (Table 3). Third, DM significantly increased the risk of gastric cancer mortality in men if the mean age was less than 55 years, the study used OR, RR, or HR as an effect estimate index, and the study was not adjusted for alcohol consumption (Table 3). There were no gender differences for gastric cancer incidence and gastric cancer mortality in specific subsets.

**Table 2:** Subgroup analysis of relative risk (ratios) for gastric cancer in men and women

Subgroup	Stratified analyses	Sex	RR and 95%CI	*P* value	I-square and *P* value for heterogeneity	RRR and 95%CI	*P* value for interaction test
Country	Western countries	Men	0.98 (0.84-1.15)	0.817	73.3% (0.005)	0.92 (0.75-1.14)	0.466
		Women	1.06 (0.92-1.22)	0.400	0.0% (0.456)		
	Eastern countries	Men	1.02 (0.86-1.20)	0.817	46.3% (0.097)	0.97 (0.72-1.32)	0.851
		Women	1.05 (0.82-1.36)	0.690	55.0% (0.049)		
Age at baseline	≥55	Men	1.06 (0.90-1.25)	0.481	64.4% (0.015)	0.95 (0.77-1.19)	0.679
		Women	1.11 (0.96-1.28)	0.175	0.0% (0.922)		
	<55	Men	1.06 (0.93-1.22)	0.373	26.5% (0.257)	0.91 (0.67-1.24)	0.566
		Women	1.16 (0.88-1.53)	0.306	59.0 (0.087)		
Effect estimate	SIR/SMR	Men	1.20 (1.05-1.36)	0.007	0.0% (0.666)	1.09 (0.88-1.35)	0.420
		Women	1.10 (0.93-1.30)	0.264	0.0% (0.933)		
	OR, RR, or HR	Men	0.97 (0.87-1.09)	0.598	66.5% (0.002)	0.92 (0.74-1.16)	0.487
		Women	1.05 (0.87-1.28)	0.594	49.4% (0.054)		
Follow-up duration (yr)	≥15	Men	0.99 (0.61-1.61)	0.957	73.2% (0.053)	1.65 (0.38-7.21)	0.506
		Women	0.60 (0.15-2.43)	0.477	82.7% (0.016)		
	<15	Men	0.98 (0.88-1.10)	0.756	64.0 (0.005)	0.91 (0.76-1.08)	0.273
		Women	1.08 (0.95-1.24)	0.248	13.2% (0.327)		
Adjusted BMI	Yes	Men	0.98 (0.83-1.16)	0.826	42.5% (0.138)	1.02 (0.58-1.79)	0.942
		Women	0.96 (0.56-1.63)	0.870	70.3% (0.018)		
	No	Men	1.01 (0.87-1.18)	0.889	68.5% (0.007)	0.92 (0.77-1.10)	0.359
		Women	1.10 (0.99-1.21)	0.067	0.0% (0.660)		
Adjusted smoking	Yes	Men	1.03 (0.90-1.18)	0.690	27.0 (0.241)	0.93 (0.66-1.30)	0.666
		Women	1.11 (0.81-1.51)	0.509	65.4% (0.021)		
	No	Men	1.00 (0.85-1.18)	0.995	74.5% (0.001)	0.96 (0.78-1.19)	0.713
		Women	1.04 (0.91-1.18)	0.592	0.0% (0.816)		
Adjusted alcohol drinking	Yes	Men	1.00 (0.89-1.12)	0.952	61.5% (0.024)	0.90 (0.65-1.26)	0.538
		Women	1.11 (0.81-1.51)	0.509	65.4% (0.021)		
	No	Men	1.02 (0.78-1.33)	0.879	76.5% (0.002)	0.98 (0.73-1.32)	0.898
		Women	1.04 (0.91-1.18)	0.592	0.0% (0.816)		
Adjusted physical activity	Yes	Men	0.98 (0.80-1.19)	0.802	0.0% (0.498)	0.77 (0.48-1.25)	0.291
		Women	1.27 (0.82-1.97)	0.284	63.0% (0.067)		
	No	Men	1.01 (0.89-1.14)	0.913	75.6% (<0.001)	0.97 (0.81-1.17)	0.757
		Women	1.04 (0.91-1.20)	0.556	27.8% (0.216)		

**Table 3:** Subgroup analysis of relative risk (ratios) for gastric cancer mortality in men and women

Subgroup	Stratified analyses	Sex	RR and 95%CI	*P* value	I-square and *P* value for heterogeneity	RRR and 95%CI	*P* value for interaction test
Country	Western countries	Men	1.19 (0.99-1.43)	0.070	24.5% (0.264)	0.91 (0.70-1.18)	0.467
		Women	**1.31 (1.09-1.57)**	**0.004**	**0.0% (0.739)**		
	Eastern countries	Men	1.61 (0.85-3.03)	0.144	99.0% (<0.001)	1.01 (0.38-2.66)	0.980
		Women	1.59 (0.77-3.31)	0.212	97.4% (<0.001)		
Age at baseline	≥55	Men	1.04 (0.84-1.28)	0.737	0.0% (0.503)	0.85 (0.61-1.20)	0.359
		Women	1.22 (0.93-1.59)	0.151	0.0% (0.773)		
	<55	Men	**1.16 (1.05-1.29)**	**0.004**	**0.0% (0.834)**	1.06 (0.84-1.35)	0.612
		Women	1.09 (0.88-1.36)	0.438	-		
Effect estimate	SIR/SMR	Men	1.65 (0.88-3.11)	0.120	90.2% (0.001)	0.98 (0.39-2.46)	0.959
		Women	1.69 (0.86-3.32)	0.127	87.9% (0.004)		
	OR, RR, or HR	Men	**1.17 (1.04-1.33)**	**0.012**	**25.9% (0.257)**	0.96 (0.79-1.17)	0.674
		Women	**1.22 (1.05-1.42)**	**0.009**	**6.4% (0.343)**		
Follow-up duration (yr)	≥15	Men	1.24 (0.67-2.29)	0.493	-	-	-
		Women	-	-	-		
	<15	Men	1.34 (0.92-1.96)	0.129	96.7% (<0.001)	0.96 (0.56-1.64)	0.874
		Women	1.40 (0.95-2.06)	0.085	97.1% (<0.001)		
Adjusted BMI	Yes	Men	1.04 (0.84-1.28)	0.737	0.0% (0.503)	0.85 (0.61-1.20)	0.359
		Women	1.22 (0.93-1.59)	0.151	0.0% (0.773)		
	No	Men	1.48 (0.96-2.28)	0.075	97.1% (<0.001)	0.97 (0.50-1.88)	0.922
		Women	1.53 (0.92-2.53)	0.100	95.7% (<0.001)		
Adjusted smoking	Yes	Men	**1.14 (1.03-1.25)**	**0.008**	**0.0% (0.498)**	1.00 (0.82-1.23)	1.00
		Women	1.14 (0.95-1.36)	0.165	0.0% (0.494)		
	No	Men	**1.58 (1.04-2.39)**	**0.032**	**91.1% (<0.001)**	0.99 (0.54-1.82)	0.968
		Women	**1.60 (1.02-2.50)**	**0.039**	**90.5% (<0.001)**		
Adjusted alcohol drinking	Yes	Men	1.12 (0.98-1.27)	0.095	23.9% (0.252)	0.98 (0.79-1.23)	0.875
		Women	1.14 (0.95-1.36)	0.165	0.0% (0.494)		
	No	Men	**1.51 (1.04-2.21)**	**0.031**	**88.1% (<0.001)**	0.94 (0.53-1.70)	0.846
		Women	**1.60 (1.02-2.50)**	**0.039**	**90.5% (<0.001)**		
Adjusted physical activity	Yes	Men	1.02 (0.81-1.29)	0.853	0.0% (0.506)	0.82 (0.55-1.22)	0.320
		Women	1.25 (0.90-1.73)	0.181	-		
	No	Men	1.45 (0.95-2.20)	0.085	97.1% (<0.001)	1.01 (0.55-1.86)	0.982
		Women	1.44 (0.92-2.24)	0.108	94.2% (<0.001)		

### Publication bias

**Figure 5:**
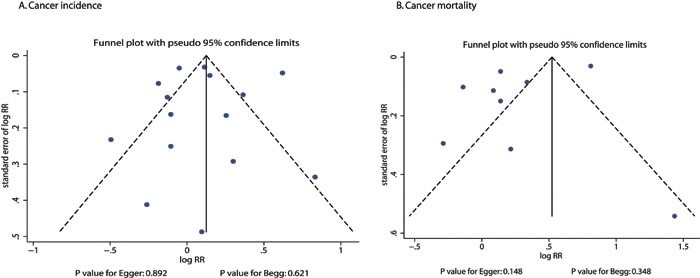
Funnel plots for gastric cancer incidence (**A**) and gastric cancer mortality (B).

Review of the funnel plots could not rule out the potential for publication bias for gastric cancer incidence and gastric cancer mortality (Figure 5). The Egger [47] and Begg tests [48] results showed no evidence of publication bias for gastric cancer incidence (*P* value for Egger: 0.892; *P* value for Begg: 0.621) or gastric cancer mortality (*P* value for Egger: 0.148; *P* value for Begg: 0.348).

## DISCUSSION

Our current study was based on cohort studies and explored all possible correlations between DM and the outcomes of gastric cancer incidence and gastric cancer mortality. This large quantitative study included 8,559,861 participants from 22 cohort studies with a broad range of populations. The findings from our study indicate that DM has no overall significant impact on the risk of gastric cancer incidence and gastric cancer mortality. Subgroup analyses suggested mean age at baseline, effect estimate, adjusted smoking, alcohol drinking or not might affect the incidence of gastric cancer mortality in men, and Country, effect estimate, adjusted smoking, alcohol drinking or not were affect gastric cancer mortality in women. However, there were no gender differences between men and women for any correlations of DM and gastric cancer.

The methodological evaluation of each included study was limited by the representativeness of the exposed cohort, selection of the non-exposed cohort, ascertainment of DM, demonstration that outcomes were not present at the start of study, comparability on the basis of the design or analysis, assessment of outcome, adequate follow-up duration, and adequate follow-up rate. Our meta-analysis of cohort studies provides unclear results for the selection of the non-exposed cohort if the study reported SIR/SMR as the effect estimate, which contributed to heterogeneity in overall analysis. Therefore, the summary results might be biased due to different effect estimate indexes.

A previous meta-analysis suggested that DM patients had a similar risk of gastric cancer incidence and substantial heterogeneity was observed. Furthermore, subgroup analyses indicated DM significantly increased the risk of gastric cancer in men, whereas it had no effect in women [23]. However, another meta-analysis suggested that total participants with DM have an increased risk of gastric cancer, and are positively associated with gastric cancer mortality [22]. The inherent limitation of those previous meta-analyses is that case control studies were included and various confounding factors might be biasing the results, as several important confounders cannot be adjusted. We therefore conducted this study to evaluate the relationship between DM and the risk of gastric cancer incidence or mortality on the basis of gender.

Most of our findings were in agreement with a recently published large cohort study conducted in the UK [54]. This prospective study included 28,900 patients with insulin-treated diabetes and found that DM was not associated with gastric cancer incidence or mortality risk. The reason for this could be that the study design used total cancer events as primary outcomes, and the sample size might not have been sufficient to evaluate the relationship between DM and gastric cancer risk. Event rates were lower than expected, which always requires broad confidence intervals, resulting in no statistically significant difference. Chodick et al [51] conducted a cohort study and, after an 8 year follow-up, concluded that there was no significant increase in overall risk of gastric cancer incidence between DM and non-DM participants. Xu et al [63] did not find any evidence that type 2 DM was associated with an increased risk of gastric cancer either in men or in women. Our current study also indicated that DM has no significant effect on the overall risk of gastric cancer. Yet symptoms of gastric cancer can be hidden and diagnosis might come late in DM patients with gastric cancer, which could incorrectly lend toward this conclusion of non-significant correlations.

There was no significant difference between DM and non-DM participants and the risk of gastric cancer incidence or mortality. However, several studies included in our study reported inconsistent results. Jee et al [16] indicated that elevated fasting serum glucose and DM are independent risk factors for gastric cancer, and the relative risk tends to increase accompanying an increased fasting serum glucose level. Similarly, Lin et al [18] found a significant association between DM and the higher risk of gastric cancer. They explained this relationship saying that DM patients with hyperglycemia may cause dysregulation of energy balance, which could affect intracellular metabolism and impair immune system and might play an important role in the progression of gastric cancer [64]. Conversely, a significantly reduced risk for gastric cancer following DM was detected in Khan et al and Adami et al [20, 21]. These could be due to time effects in certain cases, with an early decrease followed by an increased risk.

Subgroup analysis suggested that males with DM were associated with an increased risk of gastric cancer if the study used SIR/SMR as an effect estimate index. Furthermore, participants with DM might have an increased risk of gastric cancer mortality in multiple subsets. It is possible that longer diabetes duration could be associated with insulin resistance and hyperinsulinemia, which might has an effect on promoting cell growth and proliferation. Although an important population-based study compared the incidence of gastric cancer in insulin users and nonusers and showed a lack of association between insulin use and gastric cancer, which might due to DM patients received insulin or not with different DM status [65]. Furthermore, most confounders cannot be adjusted in several studies, which might bias the summary result. Finally, several conclusions may be variable since smaller cohorts were included. Therefore, relative results with a comprehensive review were provided in our study.

We noted higher heterogeneity for the summary results, the reason for this could be the baseline characteristics might affect the relationship between DM and gastric cancer incidence or mortality. Tseng et al indicated hyperglycemia, Helicobacter pylori (HP) infection, high salt intake, medications and comorbidities might play an important role on the risk of gastric cancer. They stated DM patients was associated with higher infection rate, lower eradication rate and higher reinfection rate of HP. Further, salt intake might affect HP infection [66]. However, salt intake and HP infection status were not reported in the studies included in our meta-analysis. In addition, patients in different ethnicities and geographical regions with different levels of salt intake and HP infection, which may affect the relationship between DM and gastric cancer and contribute to the potential heterogeneity. Furthermore, Tseng et al resulted DM patients received metformin might affect the incidence of gastric cancer [67]. Finally, they indicated DM was contributed a harmful effect on gastric cancer, whereas insulin use has no significant effect on the gastric cancer risk [65]. In this study, mostly studies included could not adjusted antidiabetic drugs, which may introduce potential heterogeneity.

Three strengths of our study should be highlighted. First, only cohort studies were included, which should eliminate uncontrolled biases. Second, the large sample size allowed us to quantitatively assess the association of DM with the risk of gastric cancer and mortality, potentially making our findings more robust than those of any individual study. Third, the summary RRR was employed to evaluate gender differences for this relationship.

The limitations of our study are as follows: (1) the adjusted models are different across the included studies, and these factors might play an important role in the development of gastric cancer; (2) the incidence of gastric cancer and mortality was difference, which might introduce uncontrol biases; (3) postmenopausal status in women might affect the incidence of gastric cancer or mortality, whereas the results of stratified analysis in individual study was not available; (4) in a meta-analysis of published studies, publication bias is an inevitable problem; and (5) the analysis used pooled data (individual data were not available), which restricted us from performing a more detailed relevant analysis and obtaining more comprehensive results.

The findings of this study suggest that DM is not associated with overall changes in gastric cancer or mortality risk. Furthermore, subgroup analyses suggested that certain participants (mean age at baseline less than 55.0, used RR or HR as effect estimate, the study adjusted smoking or not, and the study not adjusted alcohol drinking in men, and the study conducted in Western Countries, used RR or HR as effect estimate, not adjusted smoking or alcohol drinking in women.) with DM may see a higher risk of gastric cancer mortality. Future studies are needed to focus on specific populations and evaluate potential interactions of other important confounders.

## MATERIALS AND METHODS

We followed Preferred Reporting Items for Systematic reviews and Meta-analysis guideline in reporting this systematic review and meta-analysis [38].

### Data Sources, Search Strategy, and Selection Criteria

Literature research was carried out by searching relevant publications via the electronic databases PubMed, EmBase and the Cochrane Library. Any cohort study that examined the relationship between DM and the risk of gastric cancer incidence or mortality was eligible for inclusion in our study, and no restrictions were placed on language or publication status (published, in press, or in progress). The following search terms (“gastric” OR “stomach”) AND (“carcinoma” OR “cancer” OR “neoplasm” OR “adenocarcinoma”) AND (“diabetes” OR “diabetes mellitus”) were searched (from inception to June 2016). The details of the search strategy are listed in Supplemental 1. Manual searches of the reference lists from all the relevant studies and review articles were conducted as well. The medical subject heading, methods, population status, design, exposure, and outcome variables of these articles were used to identify the relevant studies.

The literature search was independently undertaken by 2 authors using a standardized approach. Any inconsistencies between these 2 authors were settled by the primary author until a consensus was reached. The criteria for eligibility of the studies were as follows: (1) the study had to have a cohort design (prospective or retrospective); (2) the study investigated the association between DM and the risk of gastric cancer incidence or mortality; and (3) the study should report effect estimates (risk ratio [RR], hazard ratio [HR], standard incidence/mortality ratio [SIR/SMR]) and 95% confidence intervals (CIs) for comparisons of participants with DM and those without DM. We used the following exclusion criteria: (1) the type of study was non-cohort design; (2) the studies evaluated other factors such that the relationship between DM and gastric cancer was not available; and (3) the publications were duplicated studies, abstracts, reviews, or the reported data from an abstract or from a meeting.

### Data collection and quality assessment

Studies were reviewed and data extracted independently by two authors using a pre-designed standard form. The following data were extracted from each study: the study group or first author's name, publication year, country, assessment of exposure, sample size, age at baseline, gender, percentage of participants overweight, gastric cancer cases, gastric cancer mortality cases, effect estimate, follow-up duration, and adjusted factors. For studies that reported several multivariable adjusted RRs, we selected the effect estimate that was maximally adjusted for potential confounders. Attempts were made to contact the authors for missing data.

The Newcastle-Ottawa Scale (NOS), which is quite comprehensive and has been partially validated for evaluating the quality of observational studies in meta-analysis, was used to evaluate methodological quality [39]. The NOS is based on the following 3 subscales: selection (4 items), comparability (1 item), and outcome (3 items). A “star system” (range, 0-9) has been developed for assessment (Table 1). The data extraction and quality assessment were conducted independently by 2 authors. Information was examined and adjudicated independently by an additional author referring to the original studies.

### Statistical analysis

We examined the relationship between DM and risk of gastric cancer incidence or mortality on the basis of the effect estimate (RR, HR, SIR/SMR) and its 95% CI published in each study. If more than one, subsets were pooled by using a fixed effect model to calculate their RRs and 95%CIs for effect estimates of each study [40]. We used the random-effects model to calculate summary RRs and 95%CIs for participants with DM versus participants without DM [41]. We probed the association between DM and gastric cancer in men and women separately. Finally, the ratios of relative risk (RRRs) and the corresponding 95%CIs were used to calculate gender difference for the relationship between DM and gastric cancer incidence or mortality [42].

Heterogeneity between studies was investigated by using I-square and Q statistic, and were regarded as significant heterogeneity if the *P* value was less than 0.10 [43, 44]. Sensitivity analyses were also conducted to evaluate the impact of individual studies by systematically removing each individual study from the meta-analysis [45]. Subgroup analyses were conducted for gastric cancer incidence and mortality in men and women on the basis of country, age at baseline, effect estimate, follow-up duration, adjusted BMI, smoking, alcohol consumption, and physical activity or lack thereof. Interaction tests for differences between men and women in subsets were also calculated [46]. Several methods were employed to check for potential publication bias, including visual inspections of funnel plots for gastric cancer incidence and gastric cancer mortality and the Egger [47] and Begg [48] tests. All reported *P* values are 2-sided, and *P* values less than 0.05 were regarded as statistically significant. Statistical analyses were performed using STATA software (version 12.0; Stata Corporation, College Station, TX, USA).

## SUPPLEMENTARY MATERIALS TABLES


