# Supplementary material for: Diabetes mellitus and the risk of gastric cancer: a meta-analysis of cohort studies
Source: Oncotarget. 2017 Mar 22;8(27):44881–92. doi: 10.18632/oncotarget.16487 (PMC5546528; doi:10.18632/oncotarget.16487)
Supplement: Supplementary file 1 [file oncotarget-08-44881-s001.docx]

Table S1. Sensitivity analyses for gastric cancer incidence in men

| **Excluding study** | **RR and 95%CI** | **P value** | **Heterogeneity (%)** | **P value for heterogeneity** |
| --- | --- | --- | --- | --- |
| Wideroff 1997 | 0.97 (0.87-1.08) | 0.589 | 62.4 | 0.004 |
| NHIC 2005 | 0.98 (0.86-1.11) | 0.718 | 59.9 | 0.008 |
| JPHC 2006 | 0.99 (0.89-1.11) | 0.923 | 70.1 | <0.001 |
| MHS 2010 | 0.98 (0.88-1.09) | 0.743 | 66.9 | 0.001 |
| NIH-AARP Diet and Health Study 2011 | 1.00 (0.90-1.12) | 0.999 | 70.3 | <0.001 |
| U.S. Veterans 2010 | 1.01 (0.89-1.15) | 0.880 | 60.6 | 0.007 |
| JACC 2006 | 1.01 (0.91-1.13) | 0.789 | 68.3 | 0.001 |
| Ragozzino 1982 | 1.00 (0.90-1.12) | 0.970 | 70.3 | <0.001 |
| Adami 1991 | 1.04 (0.94-1.15) | 0.463 | 60.1 | 0.007 |
| NHIRD 2013 | 1.01 (0.91-1.13) | 0.821 | 69.1 | 0.001 |
| Xu 2015 | 1.02 (0.91-1.13) | 0.786 | 68.8 | 0.001 |

Table S2. Sensitivity analyses for gastric cancer incidence in women

| **Excluding study** | **RR and 95%CI** | **P value** | **Heterogeneity (%)** | **P value for heterogeneity** |
| --- | --- | --- | --- | --- |
| Wideroff 1997 | 1.05 (0.88-1.27) | 0.577 | 42.1 | 0.086 |
| NHIC 2005 | 1.04 (0.87-1.25) | 0.664 | 38.0 | 0.116 |
| JPHC 2006 | 1.05 (0.93-1.18) | 0.462 | 21.5 | 0.251 |
| MHS 2010 | 1.07 (0.92-1.24) | 0.384 | 42.0 | 0.087 |
| NIH-AARP Diet and Health Study 2011 | 1.05 (0.91-1.21) | 0.533 | 38.4 | 0.112 |
| JACC 2006 | 1.09 (0.99-1.20) | 0.066 | 0.9 | 0.426 |
| Ragozzino 1982 | 1.07 (0.92-1.23) | 0.396 | 42.3 | 0.085 |
| Adami 1991 | 1.10 (0.95-1.28) | 0.217 | 32.5 | 0.158 |
| NHIRD 2013 | 1.07 (0.92-1.24) | 0.409 | 42.2 | 0.086 |
| Xu 2015 | 1.09 (0.93-1.27) | 0.274 | 36.9 | 0.124 |

Table S3. Sensitivity analyses for gastric cancer mortality in men

| **Excluding study** | **RR and 95%CI** | **P value** | **Heterogeneity (%)** | **P value for heterogeneity** |
| --- | --- | --- | --- | --- |
| CPS II 2004 | 1.41 (0.96-2.08) | 0.077 | 96.2 | <0.001 |
| NHIC 2005 | 1.37 (0.92-2.04) | 0.123 | 92.8 | <0.001 |
| Tseng 2011 | 1.17 (1.07-1.28) | <0.001 | 1.2 | 0.400 |
| Verona Diabetes Study 2003 | 1.36 (0.92-2.01) | 0.119 | 96.7 | <0.001 |
| Koskinen 1998 | 1.31 (0.87-1.98) | 0.197 | 96.7 | <0.001 |
| Whitehall study 2004 | 1.34 (0.92-1.96) | 0.129 | 96.7 | <0.001 |

Table S4. Sensitivity analyses for gastric cancer mortality in women

| **Excluding study** | **RR and 95%CI** | **P value** | **Heterogeneity (%)** | **P value for heterogeneity** |
| --- | --- | --- | --- | --- |
| CPS II 2004 | 1.44 (0.92-2.24) | 0.108 | 94.2 | <0.001 |
| NHIC 2005 | 1.51 (1.02-2.23) | 0.040 | 90.1 | <0.001 |
| Tseng 2011 | 1.21 (1.06-1.40) | 0.006 | 0.0 | 0.533 |
| Verona Diabetes Study 2003 | 1.46 (0.95-2.23) | 0.082 | 94.4 | <0.001 |
| Koskinen 1998 | 1.40 (0.87-2.26) | 0.170 | 94.2 | <0.001 |
